# The therapeutic importance of understanding mechanisms of neuronal cell death in neurodegenerative disease

**DOI:** 10.1186/1750-1326-4-8

**Published:** 2009-02-04

**Authors:** Todd E Golde

**Affiliations:** 1 Department of Neuroscience, Mayo Clinic, Mayo Clinic College of Medicine, 4500 San Pablo Road, Jacksonville, Florida 32224, USA

## Abstract

Despite major advances in our understanding of the initiating factors that trigger many neurodegenerative disorders, to date, no novel disease-modifying therapies have been shown to provide significant benefit for patients who suffer from these devastating disorders. As most neurodegenerative disorders are late-onset, slowly progressive, and appear to have long relatively asymptomatic prodromal phases, it is possible that therapies optimally targeting the triggers of these disorders may have limited benefit when treatment is initiated in the symptomatic patient. Such therapies may work in the prodromal phase, or when given prophylactically, but in the symptomatic patient there simply may be too much damage to the neuronal networks to restore functionality by reducing or even eliminating the primary stressor. As functional neuronal demise and overt neuronal death are almost certainly the key factors that mediate the functional impairment, it is clear that preventing neuronal death and dysfunction will have a huge clinical benefit. Unfortunately, we lack a detailed understanding of neuronal death pathways in almost all neurodegenerative disorders. To rationally develop new disease modifying therapies that target steps in the degenerative cascade downstream of the disease trigger will require a number of factors. First, we need to refocus our basic research efforts on identifying the precise steps in the pathological cascade that lead to neuronal death in each neurodegenerative disease and, if possible, determine the relative placement of those events within a potentially very complex cascade. Second, we will need to determine which of these steps are potentially targetable. Finally, we will need to develop novel therapies that interfere with these steps and demonstrate that such therapies alone, or in combination with therapies that target the trigger of these devastating diseases, have clinical benefit.

## Introduction

Over the last two decades there has been enormous progress with respect to understanding the initiating factors that trigger complex cascades that ultimately result in various neurodegenerative disorders [1-3]. Much of this progress has been the result of 1) biochemical and histochemical characterization of proteins that accumulate within various inclusions in the diseased brain and 2) genetic linkage studies identifying mutations in genes that cause neurodegenerative disease. These biochemical, histochemical, and genetic studies were often mutually reinforcing; the protein found to accumulate in the brain proved often to be the same protein encoded by the disease causing mutant gene (see Table 1). Once mutant genes have been identified, animal models that recapitulate at least some aspects of the human disease have been made by creating transgenic mice expressing the mutant genes in the brain [4,5]. As there are no naturally occurring animal models for many neurodegenerative diseases, the creation of animal and cell based models based on mutant genes identified in humans have had a tremendous impact on target identification, enabled pre-clinical studies of novel therapies in those animal models, and led to the clinical testing of a number of novel therapeutics. Indeed, the advent of animal models that recapitulate features of Alzheimer' disease (AD), Parkinson's disease (PD), Huntington's disease (HD), familial amyotrophic lateral sclerosis (ALS), and host of other human neurodegenerative diseases, has catalyzed efforts to develop therapies aimed at modifying the underlying disease process and, to some degree, redirected the field from developing symptomatic therapies to these potential disease-modifying therapies.

**Table 1:** Examples of the Relationship Between Lesions, Proteins and Causal Genes in Neurodegenerative Diseases

**Disease**	**Lesions**	**Major Protein In Deposit**	**Genetic Alterations**
Alzheimer's	Plaques NFT	Aβ tau	Mutations/Multiplication in APP and Presenilin genes cause altered Aβ production

Parkinson's (classic)^1^	Lewy Body	α-synuclein	Mutation/Multiplication of α-synuclein gene

FTDP-17tau	NFT	tau	Mutations in tau

FTLDu	Ubiquitin positive inclusions	TDP-43	Mutations in Granulin gene, evidence that Granulin interacts with TDP-43

Polyglutamine Diseases (e.g. Huntington's, SCA)	Inclusions of expanded Poly Q protein	PolyQ encoding protein	Expanded CAG repeats encoded stretches of Poly Q

ALS	Ubiquitin positive inclusions	TDP-43	Mutations in TDP-43

ALS	Ubiquitin positive inclusions	Inclusions are TDP negative	Mutations in SOD1

^1 ^Numerous genes have now been implicated in Parkinsonism, some of these genes result in classic Lewy body pathology others do not, and some such as LRRK2 can produce distinct pathologies even within a single family.

Despite this progress in identifying the trigger of many neurodegenerative diseases there have been only limited advances with respect to the clinical treatment of patients who suffer from these devastating and, for the most part, lethal diseases. To date, these advances have stemmed not from understanding the trigger of the disease, but largely from understanding the neurochemical and functional circuitry deficits present. For example the development of cholinesterase inhibitors for the treatment of AD was largely based on the observation that in AD cholinergic neurons are severely depleted and cholinesterase and choline acetyl transferase activity are markedly reduced compared to controls. Indeed the neurochemical deficits that have been reported are striking, with reductions of up to 90% activity in AChE and CHAT in postmortem brains of AD patients [6-8]. Similarly, the development of levodopa therapy was based on the reduction in dopamine levels in the basal ganglia [9-11]. Indeed, it is well established that gross motor impairments are not present in most patients with Parkinson's disease until > 70–80% of the dopaminergic neurons in the substantia nigra have degenerated or died, with accompanying loss of the dopaminergic input from the nigra to the striatum.

Notably, these symptomatic therapies appear to have little, if any, disease-modifying effect; though symptoms may be improved, the inevitable progression of the disease is not altered to any great extent. Obviously, a therapy that provides some symptomatic relief is better than no therapy at all, which is what is available for almost all of the uncommon neurodegenerative conditions. However, ideally, we would like to do better and not only improve symptoms but markedly slow or halt the progression of neurodegeneration.

### The natural history of typical neurodegenerative diseases and prophylactic therapy

**Figure 1:**
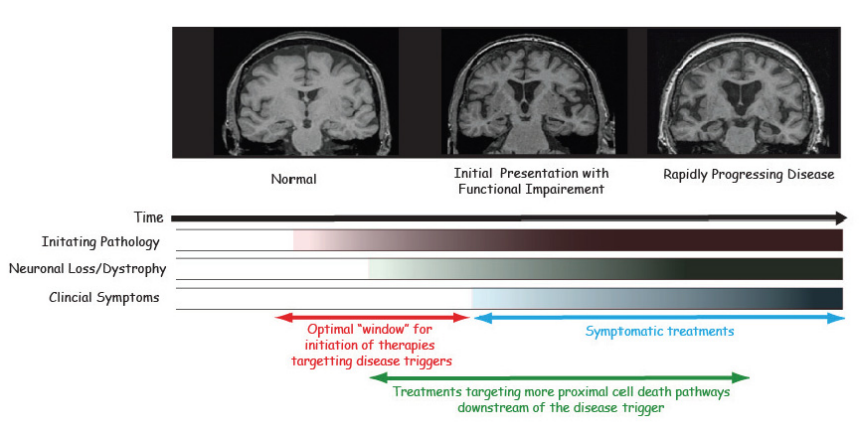
**Progression of a typical Neurodegenerative Disease**. Alzheimer's disease is used as an example to exhibit the natural progression of a neurodegenerative disease. In the top panel typical magnetic resonance imaging (MRI) scans are shown from a cognitively intact "normal" subject (left), a subject with mild cognitive impairment of the Alzheimer's type (middle), and a patient with AD (right). Note that even the subject with MCI despite displaying minimal symptoms has already clearly lost brain mass and that this increases as the disease progresses. Indeed there is a clear cascade effect in which underlying pathology drives neuronal loss and degeneration leading to clinical symptoms. Based on this one would argue that the optimal time to target the "trigger" of AD would be prior to any signs of damage to the brain or during the initial prodromal phase when pathological changes might be apparent but no clinical signs are yet apparent. Another therapeutic opportunity would be to try to stop the neuronal loss downstream of the initiating pathology.

Based on the study of patients with genetic forms of common and uncommon neurodegenerative diseases it can be inferred that the majority of these diseases feature a fairly long disease free period in which no pathological or clinical changes are noted. Subsequently, there appears to be a prodromal phase where the initiating pathology is detectable, but the patient remains symptom free. This prodromal phase may last for years or even decades. During this phase additional pathological changes occur in the brain, including neuronal loss, dystrophy, and synaptic dysfunction. Thus, when symptoms become apparent there is typically extensive damage to either vulnerable brain regions in diseases that are characterized by more focal dysfunction or widespread damage to multiple brain regions. Indeed, given compensatory mechanisms, neuronal reserve, and the relatively insensitive tests used for clinical diagnosis of most neurodegenerative conditions, by the time a patient presents with symptoms there is extensive neuronal loss and dystrophy. Such a natural progression is characteristic of Alzheimer's disease (Figure 1), where brain atrophy can be seen even in patients with mild-cognitive impairment of the AD type, a well-recognized prodromal phase of AD. Notably, if they come to autopsy, patients with MCI of the AD type also exhibit extensive AD neuropathology, and cross-sectional study of postmortem brains from patients with Trisomy 21 indicate that these individuals will become demented long after they show the initial signs of AD pathology, namely accumulation of amyloid beta protein (Aβ) in plaques and tau in neurofibrillary tangles. Although difficult to prove definitively, one can infer, based on the imaging studies, that one can lose up to 80% of their dopaminergic neurons in the substantia nigra before showing overt motor deficits [12,13]. Indeed, the latter example suggests that symptoms may result from the additional loss or dysfunction of relatively few surviving neurons, whereas the massive loss of neurons in the prodromal disease phase did not result in overt clinical symptoms.

If massive neuronal loss or massive neuronal dysfunction occurs before overt symptoms, and there is a long delay between initiating pathology and the clinical symptoms, it is only reasonable to question the potential "therapeutic" effectiveness of treatments targeting the trigger of the disease. In contrast, it is reasonable to propose that such treatments would be highly effective, if initiated prior to onset of the primary pathology or during the early phases of that pathology prior to significant neuronal loss. The problem with such a scenario is that it may take decades to prove that a given therapy works, and given the financial, regulatory, and ethical obstacles to such true prevention trials, there is no current road map that suggests such prevention trials will become feasible in the near future. Thus, investigators who study neurodegenerative diseases and are serious about translating an enhanced understanding of pathogenesis of these diseases into therapies face a very challenging paradox: most therapies developed as potentially disease modifying target the trigger of the disease; yet they will be tested in the therapeutic setting where it is not unreasonable to think they will be ineffective or minimally effective. There simply has been too much downstream damage to the brain.

### Pathogenic cascades downstream of disease triggers

Despite the progress in understanding the triggers of neurodegenerative disease, the precise pathways that lead to neuronal death are not well established. Indeed, for almost every neurodegenerative disease multiple hypotheses exist regarding the mechanism or mechanisms that lead to neuronal dysfunction. In many cases, common pathways are invoked across multiple diseases. Indeed, inflammation, oxidative stress, mitochondrial dysfunction, and axonal transport deficits are postulated to play some role in almost every neurodegenerative disorder [14-18]. Likewise, proteasomal dysfunction, chronic stress responses, altered autophagy, calcium dyshomeostasis, and neuronal cell cycle induction also appear to be common, if not universal features, of many neurodegenerative diseases [19-25]. However, placing these pathways in the proper relationship to the disease trigger is challenging.

**Figure 2:**
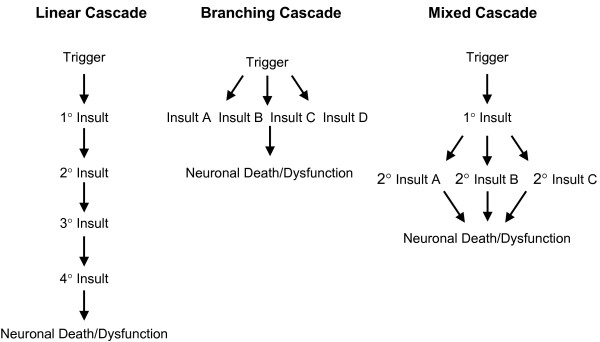
**Different types of Neurodegenerative Cascades**. The utility of targeting events downstream of any disease trigger will in part be determined by the type of cascade that is established. If a linear cascade (left panel) is established there may be many events that can be targeted that will have equal efficacy. Halting any step in the cascade may stop the cascade. In a branching cascade (middle panel), the situation where a single initiating event triggers multiple insults that then mutually contribute to neuronal death, the efficacy of targeting a single downstream pathway may be much more limited. In the case of a mixed cascade (right panel), in which an initiating event can trigger a complex cascade which may initially be linear and then later branch, understanding the placement of any given insult within cascade may be critical with respect to determining how effective therapies targeting that step will be with respect to slowing or halting neurodegeneration.

From a completely conceptual point of view, one can think of the pathological cascades initiated by a given neurodegenerative disease trigger in three different generic scenarios (Figure 2). The first scenario is a completely linear cascade in which the initial toxic insult leads to a linear cascade that eventually results in neuronal death. The second scenario would be a branching cascade where the toxic insult simultaneously initiates multiple pathological cascades that then collectively contribute to neuronal death and dysfunction. The third scenario is a mixed cascade where both linear and branching cascades might be initiated in various orders by the trigger.

The distinctions between these types of cascades are not trivial. From an investigational point of view, it has proven extremely challenging to precisely order the events in the cascade even in animal models that show massive neuronal loss, such as mutant tau models of FTDP-17 [26]. From a therapeutic point of view distinguishing between these cascades will almost certainly guide decisions regarding therapies that target factors downstream of the trigger. For example, if neuronal death is mediated by a linear cascade, the likelihood that a therapeutic agent targeting any one of the insults within that cascade will have a major effect is much larger. This is especially true if the therapeutic agent is targeting an early insult in the cascade. If neuronal death is mediated by a branching cascade, a therapy targeting any single downstream pathway may have limited efficacy. In any case, given that in most neurodegenerative diseases a mixture of linear and branching cascades are likely to play a role in neuronal death, enhanced efforts to understand the order of events would seem to be critical in order to efficiently guide optimal therapies.

### How do we dissect the downstream cascades?

Even with advances in imaging the human brain and development of probes for certain molecular phenotypes (e.g. amyloid imaging agents to visualize amyloid load in AD), it is unlikely that study of the progression of human neurodegenerative disease will be sufficient to dissect the downstream cascades leading to neuronal death. Likewise, cell culture models of neurodegenerative disease may provide hypotheses regarding the primacy of various pathways, but such hypotheses eventually will need to be tested in animal models. Indeed, animal models of neurodegenerative disease are essential to developing novel therapies and understanding the role of various factors in driving neuronal death and dystrophy. However, one must be cautious about "therapeutic" studies in animal models. In many cases the studies are not therapeutic but actually reflect primary prevention. The treatment was initiated prior to the onset of detectable pathology. In other cases, such as many APP mouse models of Aβ deposition, the mutant human transgene expression in the brain does not fully recapitulate the human disease, especially the extent and degree of neuronal loss. In such cases one has to question whether a treatment that shows efficacy in the mouse model with no neuronal loss will be effective in a human with massive neuronal loss.

### Should the field refocus on the mediators of cell death and therapies targeting those pathways?

There is a sharp dichotomy within the neurodegenerative disease field between common diseases such as AD and PD and the plethora of rare neurodegenerative disease such as the polyglutamine disorders, with respect to commercial interest in developing novel therapies. For the common neurodegenerative conditions, there is tremendous incentive to develop new therapies. There is a huge unmet medical need, a huge market, and a new AD or PD therapy even of modest efficacy will be a blockbuster in terms of sales. In contrast, the relatively small number of patients with any individual rare neurodegenerative diseases means that there is much less commercial interest in developing new therapies. Thus, most therapeutic development for these conditions occurs in academic laboratories with an increasing effort from philanthropic organizations to assist with the clinical development. Occasionally, biotech companies or major pharmaceutical companies will partner in these efforts, but typically with limited resources.

If one considers the recent therapeutic developments in AD as an example of the prioritization of therapeutic development in a common neurodegenerative disease, one can see that pharmaceutical companies have largely invested in two main strategies. Targeting the trigger, namely efforts to prevent, remove, or neutralize accumulation and aggregation of toxic Aβ; and, more recently, a renewed interest in cognitive-enhancing agents. There is very limited development of novel strategies intervening downstream of the trigger, largely because there is not a good "road map" for what to target. Yet, based on rationale outlined above, therapies targeting downstream triggers could have a major impact on disease progression.

With the downsizing of basic research efforts within the commercial sector, it is highly likely that most novel targets downstream of the trigger will be identified in the non-profit sector. However, again using AD as an example, much of the funding portfolio for basic AD research is invested in factors that influence the trigger, and a much smaller segment of the portfolio is focused on those downstream pathways. As one could argue, at least with respect to anti-Aβ therapies, that the numbers of therapeutic approaches are sufficient to determine whether such strategies will be efficacious in humans with AD, it would seem that there should be a renewed focus by academics with respect to identifying the downstream pathways that mediate neuronal death. Such studies will almost certainly identify novel targets that then can be moved towards the clinic.

In contrast to diseases where there is a clear commercial incentive for therapeutic development (e.g. AD and PD), the non-profit sector must play a larger role in therapeutic development for the uncommon neurodegenerative diseases. One possible way to leverage such therapeutic development is to demonstrate that the pathway targeted may actually play a role not only in the rare condition but in a common condition such as PD or AD. Obviously, this is unlikely to work for therapies targeting a trigger of the disease, but could work if dysfunction of a critical downstream pathway drives neuronal dysfunction and death in multiple diseases.

### Summary

In this review series, a panel of experts in the neurodegenerative disease field will address the questions of "What kills neurons in neurodegenerative disease?" In the vast majority of cases, we simply don't know the detailed answer. We do know the trigger, or at least in most diseases there is a reasonable consensus that we know enough about the trigger to develop strategies to target it therapeutically (Appendix 1). It is the intent of this series to provide a "road map" for the field to refocus on the question of the pathways that conspire to kill neurons in various neurodegenerative diseases, and hopefully galvanize a new era of therapeutic development that will result in novel disease modifying therapies for patients suffering form these devastating disorders (Appendix 2).

## Competing interests

The author declares that they have no competing interests.

## Appendix 1

### Key observations

• Through genetic, pathological, biochemical, and animal modeling studies the triggers of many neurodegenerative disease have been identified.

• Most neurodegenerative diseases are late-onset, slowly progressive, and appear to have long relatively asymptomatic prodromal phase.

• Clinical symptoms are typically apparent only after extensive pathological damage including extensive neuronal dystrophy and degeneration.

• The cascades downstream of the initiating triggers of disease that lead to neuronal death and dysfunction remain enigmatic for most neurodegenerative diseases.

• Therapies targeting the triggers of neurodegenerative diseases are likely to have the greatest benefit if given during the prodromal phase or earlier.

• Therapies that prevent neuronal death and dystrophy by targeting steps in the cascade downstream of the disease trigger may provide disease modification even in patients with clinical symptoms.

## Appendix 2

### Critical next steps

• Intensify efforts to indentify the pathways that lead to neurodegeneration.

• Not only identify the individual steps within the neurodegenerative cascade, but also correctly order the steps within the cascade.

• If animal models do not fully recapitulate neurodegenerative disease phenotypes continue to develop better animal models.

• Develop safe therapies that are designed to block downstream events in neurodegenerative disease cascades and prevent neuronal dysfunction and degeneration in preclinical studies.

• Test these therapies in the clinic.
